# Histopathological and clonal study of combined lobular and ductal carcinoma of the breast

**DOI:** 10.1111/pin.12065

**Published:** 2013-06-20

**Authors:** Eri Tazaki, Yukiko Shishido-Hara, Natsuko Mizutani, Sachiyo Nomura, Hirotsugu Isaka, Hiroki Ito, Kentaro Imi, Shigeru Imoto, Hiroshi Kamma

**Affiliations:** 1 Department of Surgery, Kyorin University, Graduate School of Medicine, The University of TokyoTokyo, Japan; 2 Department of Pathology, Kyorin University, School of Medicine, The University of TokyoTokyo, Japan; 3 Department of Gastrointestinal Surgery, Graduate School of Medicine, The University of TokyoTokyo, Japan

**Keywords:** breast cancer, ductal carcinoma, human androgen receptor (*HUMARA*) gene, lobular carcinoma, methylation-specific PCR

## Abstract

Lobular carcinoma in situ (LCIS) clinically constitutes a risk factor for the subsequent development of either invasive lobular carcinoma (ILC) or invasive ductal carcinoma (IDC). In order to approach the possibility of this common precursor of both ILC and IDC, we investigated combined lobular and ductal carcinomas. Thirty-two cases of lobular carcinoma were picked up out of 773 cases of operated breast carcinomas. The histopathological detailed re-examination using immunostain of E-cadherin and β-catenin revealed a rather high frequency of combined lobular carcinomas than previous reports. Clinicopathologically, combined lobular carcinomas were younger and smaller than pure lobular carcinomas, and the cytological atypia was relatively low. These results suggested that combined lobular carcinomas could be detected in the earlier stage of breast cancer. Furthermore, the lobular and ductal components of combined carcinomas coexisted in the neighborhood and were distributed contiguously. The immunohistochemical phenotypes of both components were accorded in most combined cases. A genetic analysis using methylation-specific PCR on the *HUMARA* gene demonstrated that the same allele was inactivated in both lobular and ductal components in all detectable cases of combined carcinoma. Therefore, it is reasonable to assume that both lobular and ductal components of combined carcinomas are clonal and derived from the LCIS as the common precursor lesion, which may contradict the conventional concept that the lobular and ductal carcinomas arise from distinct differentiation pathways.

Lobular and ductal carcinomas of the breast have different characteristics in morphology and proliferation manner, although both originate from the terminal ductal-lobular unit (TDLU). The lobular carcinoma lacks E-cadherin expression on cell membrane and is loosely cohesive in cell proliferation, whereas the ductal carcinoma has E-cadherin expression and shows a cohesive arrangement with a tendency of tubule formation. Precursor lesions of either type originate mostly from the TDLU.^1^–^3^ Lobular neoplasia, which rigorously comprises lobular carcinoma in situ (LCIS) and atypical lobular hyperplasia, constitutes a risk factor and a nonobligatory precursor for the subsequent development of both invasive lobular carcinoma (ILC) and invasive ductal carcinoma (IDC).^4^–^6^ In contrast, ductal intraepithelial neoplasia, which comprises ductal carcinoma in situ (DCIS) and atypical ductal hyperplasia (ADH), is clinically considered to be a risk factor and a precursor of only IDC,^7^ but not of ILC. A new revised model of progression from in situ to invasive carcinoma has been proposed based on the molecular data.^8^ It suggests that the estrogen receptor (ER)-positive and ER-negative pathways of breast carcinogenesis are fundamentally distinct, and that lobular neoplasia, mostly LCIS has the possibility to progress to low-grade DCIS as well as to classical ILC in the ER-positive pathway. ILC represents 5–15% of invasive breast carcinomas,^9^–^13^ and it is reported that 5% of ILC showed mixed ductal and lobular features.^14^ A previous study reported that seven of 10 cases of combined DCIS and LCIS displayed loss of a common allele, suggesting a clonal relationship.^15^ So it is hypothesized that lobular and ductal carcinoma coexisting in the proximity could be derived from common precursor lesions of TDLU origin, and at least a part of invasive ductal carcinoma could be derived from lobular neoplasia or LCIS. To prove this hypothesis, we studied 15 cases of coexisting of lobular and ductal carcinoma in the ipsilateral breast. Detailed histopathological analysis was performed in order to clearly define distribution of lobular and ductal components. The clonal analysis was also undertaken to clarify the genetic relationship between both components using a methylation-specific PCR technique on the human androgen receptor (*HUMARA*) gene.^16^ The *HUMARA* gene contains highly polymorphic CAG repeat and has about 90% of heterozygosity. Since either the paternal or the maternal X chromosome is randomly inactivated in normal females,^17^,^18^ polyclonal cellular population will comprise a random X chromosome inactivation pattern, contrasted with monoclonal cellular population displaying a non-random inactivation pattern. That is, this technique evaluates the cellular clonality with random or non-random X-chromosome inactivation patterns, and is adequate only for female samples.

## Materials and Methods

### Patients and tissue specimens

All samples of breast cancer that had undergone surgery between 2005 and 2009 at Kyorin University Hospital, Tokyo, Japan, were reviewed. In 773 cases of operable breast cancer patients, 32 cases (4.1%) were lobular carcinoma. Three of 32 lobular cases were excluded from the examination, due to preoperative systemic chemotherapy or hormone therapy. Hence, a total of 29 cases of lobular carcinoma were reviewed. Surgical specimens were prepared and analyzed histologically and immunohistochemically as described below. Clinicopathologic information was obtained by reviewing pathology reports, and all materials were re-examined by two experienced pathologists. The diagnostic criteria followed those of the World Health Organization.^19^ The maximum diameter of the tumor was measured histopathologically, excluding ducal spread. The study protocol was approved by the Ethics Committee on Human Research of Kyorin University and patient anonymity was preserved.

### Histological and immunohistochemical analyses

The sections (5 cm) of formalin-fixed paraffin-embedded surgical tissues were stained with hematoxylin-eosin (HE), and used for immunohistochemical analysis. Immunostaining was performed using the Envision+ system (Dako, Glostrup, Denmark), according to the manufacturer’s instruction. The primary antibodies used and the dilation condition in this studies are as follows: anti-estrogen receptor (ER) antibody (clone 1D5, Dako; 1:50), anti-progesterone receptor (PR) antibody (clone PgR 636, Dako; 1:800), anti-E-cadherin antibody (clone 36, BD Biosciences, Franklin Lakes, NJ, USA; 1:200), and anti-β-catenin antibody (clone 14, BD Biosciences; 1:1000). For detecting human epidermal growth factor receptor 2 (HER2), HercepTest kit (Dako) were used.

For evaluation of the data, ER and PR were regarded as positive when more than 1% was stained. HER2 was scored followed by the protocol of Dako: 0, no staining; 1+, weak and incomplete membranous staining in at least 10% of the tumor cells; 2+, weak to moderate, complete membranous staining in at least 10% of the tumor cells; 3+, strong, complete membranous staining in at least 30% of the tumor cells.^20^ For the purpose of estimating the phenotypic features of tumor cells, this scoring was applied for both invasive and non-invasive lesions of both lobular and ductal carcinoma.

### Methylation-specific PCR for the human androgen receptor (*HUMARA*) locus

Formalin-fixed and paraffin-embedded tissues were sliced into 10 μm-thick sections for combined lobular and ductal carcinoma cases. The DNA was prepared from carcinomatous tissues of both lobular and ductal components using a laser-beam micro-dissection (PALM microbeam IV, Robosoftware 4.2, Carl Zeiss, Munich, Germany) to avoid contamination of stromal cells. The DNA was prepared from invasive lesions in the cases with invasive carcinomas, and from non-invasive lesions in the cases only with in-situ carcinomas. The normal control DNA was also prepared from non-carcinomatous breast tissue of some combined cases. The area of each collected lesion was more than 9 mm^2^. The DNA was extracted using QIAamp DNA FFPE tissue kit, (QIAGEN, Hilden, Germany).^21^

Methylation-specific PCR was performed according to Kubota *et al*.^22^ Although a PCR-based method for the *HUMARA* locus generally uses methylation-sensitive restriction enzymes such as *Hpa*II or *Hha*I, this method is independent of the use of the enzymes, and thus free from errors due to incomplete digestion of template DNA. The extracted DNA was first treated with sodium bisulfite to convert all unmethylated cytosine to uracil in use of Epitect Bisulfite kit (QIAGEN).^23^–^25^ The PCR primer pairs, AR-M and AR-U were used, for methylated and unmethylated DNA sequences respectively, in the highly polymorphic CpG island (exon 1) of the *HUMARA* gene (GenBank accession number M35844). The details of primer sequences have been described previously^22^ and in Table 1.

**Table 1:** AR primers used for methylation-specific PCR

Name	Sequence 5′-3′
AR-Mf	GCG AGC GTA GTA TTT TTC GGC
AR-Mr	AAC CAA ATA ACC TAT AAA ACC TCT ACG
AR-Uf	GTT GTG AGT GTA GTA TTT TTT GGT
AR-Ur	CAA ATA ACC TAT AAA ACC TCT ACA

Mf, methylated forward; Mr, methylated reverse; Uf, unmethylated forward; Ur, unmethylated reverse.

The PCR reactions were carried out with 20 ng of bisulfite treated DNA and 0.5U of HotStarTaq (QIAGEN) in use of GeneAmp PCR System 9700 (Applied Biosystems, Carlsbad, CA, USA). One primer was labeled with the fluorescent dye, FAM (carboxyfluorescein). The reactions were started with an initial denaturation at 95°C for 15 min, and then followed by 45 cycles of 94°C for 30 s, 62°C for 30 s for AR-M, and 55°C for 30 s for AR-U. Final extension was carried out at 72°C for 5 min.

The PCR products were mixed with Hi-Di formamide (Applied Biosystems) and Gene Scan 500 ROX size standard (Applied Biosystems), and after heating at 95°C for 2 min, immediately chilled with ice. The products were then capillary electrophoresed in use of an ABI PRISM 3130 Genetic Analyzer (Applied Biosystems) and the peak patterns were automatically scanned by software GeneMapper 4.0 (Applied Biosystems).^26^ Statistical analysis was performed with JMP8 software (SAS Institute, Cary, NC, USA). The Wilcoxon test was used when comparing groups. A *P*-value <0.05 was considered statistically significant.

## Results

### Clinicopatholgical and hisitopathological study

**Figure 1:**
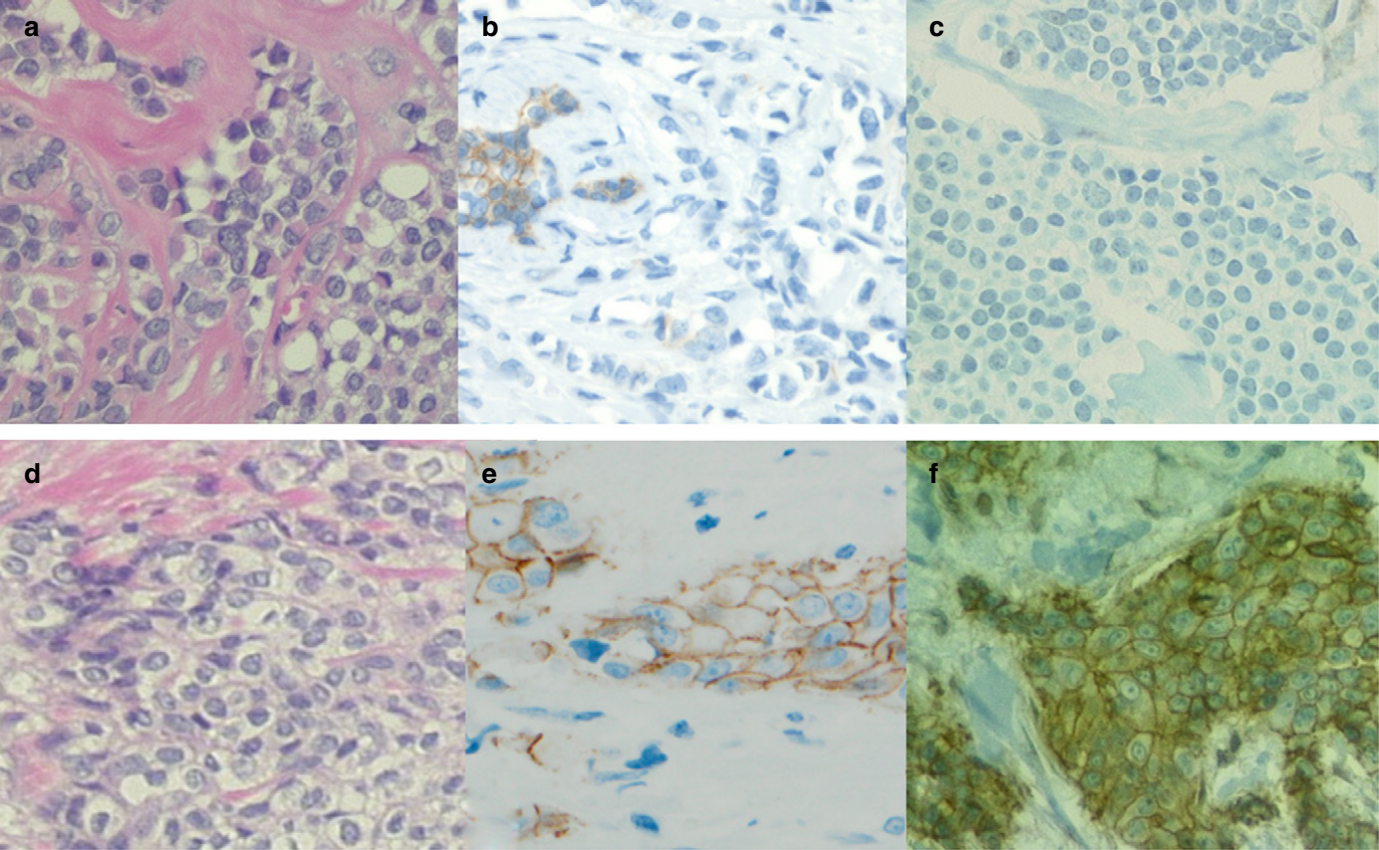
Immunohistological distinction of (a–c) lobular carcinoma and (d–f) ductal carcinoma, using (b,e) E-cadherin and (c,f) β-catenin. The lobular carcinoma cells show uniform round nuclei with sparse cytoplasm, and they are loosely cohesive each other (a, HE staining). Neither E-cadherin nor β-catenin is detected on the cell membrane, and weak-stained E-cadherin is seen in the nuclei. In contrast, ductal carcinoma cells display rather cohesive arrangement (d, HE staining), and both E-cedherin and β-catenin are distinctively expressed on the cell membrane (e,f).

Clinicopathological characteristics of 29 cases of lobular carcinoma were rearranged and histopathological re-examinations were performed using immunostain of E-cadherin and β-catenin. Representative pathological features for ILC and IDC are presented in Fig. 1. Lobular carcinoma cells were loosely cohesive without E-cadherin and β-catenin expression on the cellular membrane. In contrast, ductal carcinoma cells formed glandular structures with cohesive arrangement with distinct expression of E-cadherin and β-catenin on the cellular membrane, being consistent with previous reports.^14^,^27^,^28^

In 15 out of 29 cases (52%), coexistence of lobular and ductal carcinomas in the ipsilateral breast were exhibited, thus designated as ‘combined lobular and ductal carcinoma’. Cases where only lobular components were found were designated as ‘pure lobular’ cases. The 15 combined cases are summarized in Table 2, and divided into four groups based on the combination patterns; LCIS and DCIS for 4 cases, LCIS and IDC for 5 cases, ILC and DCIS for 5 cases, and ILC and IDC for 1 case. On the other hand, 14 cases (48%) were pure lobular carcinoma, which were all ILC (Table 3). The average age of the combined lobular cases was 52.1 years, 10 years younger than that of pure lobular cases. The average tumor size of the combined lobular cases was 2.8 cm for ILC, and 1.5 cm for IDC. In contrast, the average tumor size of the pure lobular cases measured 3.3 cm. Therefore, the clinical stage was earlier in combined lobular carcinoma cases, although there was no significant difference.

**Table 2:** Cases of combined lobular and ductal carcinoma

				Lobular component				Ductal component			
Case number	Age (year)	Clinical stage	Type	Grade	E/P/H	Size (cm)	Type	Grade	E/P/H	Size (cm)	Posional relation
1	75	0	LCIS	1	+/+/−	–	DCIS	1	+/+/−	–	C
2	44	0	LCIS	1	+/+/−	–	DCIS	1	+/+/−	–	C
3	43	0	LCIS	1	+/−/−	–	DCIS	2	+/+/−	–	C
4	50	0	LCIS	1	+/+/−	–	DCIS	2	+/+/−	–	C
5	43	I	LCIS	1	+/+/−	–	IDC	1	+/+/−	0.4	C
6	47	I	LCIS	1	+/+/−	–	IDC	1	+/+/−	1.2	C
7	50	I	LCIS	1	+/+/−	–	IDC	1	+/+/−	1.3	C
8	35	I	LCIS	1	+/+/−	–	IDC^*^	2	+/+/−	1.4	C
9	42	IIA	LCIS	1	+/+/−	–	IDC	2	+/+/−	1	C
10	43	I	ILC	2	+/+/−	1.5	DCIS	1	+/+/−	–	C
11	53	I	ILC	1	+/+/−	1.5	DCIS	2	+/+/−	–	S
12	58	IIA	ILC	2	+/−/−	2.2	DCIS	2	+/−/−	–	C
13	75	IIB	ILC	2	+/−/−	3.5	DCIS	2	+/−/−	–	C
14	73	IIA	ILC	1	+/+/−	4	DCIS	2	+/+/−	–	C
15	51	IIA	ILC	1	+/+/−	4	IDC	2	+/+/−	3.5	U
Average	52.1	–	–	–	–	2.8	–	–	–	1.5	–

C, contiguous; DCIS, ductal carcinoma in situ; E, estrogen receptor; H, human epidermal growth factor receptor 2(HER2); IDC^*^, IDC + DCIS; IDC, invasive ductal carcinoma; ILC, invasive lobular carcinoma; LCIS, lobular carcinoma in situ; P, progesterone receptor; S, separate; U, unknown.

**Table 3:** Cases of lobular carcinoma without ductal carcinoma

				Lobular component		
Case number	Age (year)	Clinical stage	Type	Grade	E/P/H	Size (cm)
1	62	I	ILC	2	+/−/−	1
2	50	I	ILC	1	+/+/−	1
3	42	I	ILC	1	+/+/−	1.5
4	79	I	ILC	3	+/−/−	1.7
5	77	I	ILC	1	+/−/−	1.9
6	72	I	ILC	1	+/−/−	1.9
7	76	IIA	ILC	1	+/+/−	2.4
8	59	IIA	ILC	1	+/+/−	3
9	74	IIA	ILC	2	+/−/−	3
10	77	IIA	ILC	1	+/+/−	4
11	36	IIB	ILC	1	+/+/−	4
12	92	IIB	ILC	1	+/+/−	5.3
13	43	IIB	ILC	1	+/+/−	7
14	49	IIIA	ILC	1	+/+/−	8
Average	63.4	–	–	–	–	3.3

E, estrogen receptor; H, human epidermal growth factor receptor 2 (HER2); ILC, invasive lobular carcinoma; P, progesterone receptor.

The cytological atypia of the lobular and ductal components were classified into three grades based on the nuclear pleomorphism and mitotic count, using the Nottingham grading system^29^ (Tables 2,3). In combined cases the cytological atypia of LCIS components were all classified into grade 1, and those of DCIS, ILC and IDC components were classified in grade 1 and 2. In pure lobular cases, the cytological atypia were variable and grade 3, which corresponded to pleomorphic lobular carcinoma, was included. Immunohistochemically, all the cases of combined lobular and ductal carcinoma expressed ER but not HER2 (Table 2). The immunohistochemical phenotypes of lobular and ductal components fully coincided and were classified as luminal A type.

**Figure 2:**
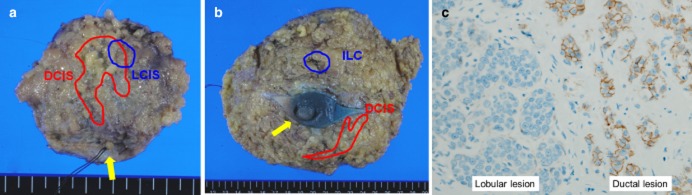
Positional relationships of lobular carcinoma component (blue) and ductal carcinoma component (red) in combined cases. (a) The contiguous distribution of lobular and ductal components in a partially-resected breast. LCIS (lobular carcinoma in situ) and DCIS (ductal carcinoma in situ) coexist in the neighborhood within the same side from the nipple (yellow arrow). (b) The separate distribution of lobular and ductal components in a totally-resected breast. ILC (invasive lobular carcinoma) and DCIS are located separately in the opposite sides across the nipple (yellow arrow). (c) Microscopic photo of Case no. 4, in which lobular and ductal components are distributed contiguously.

To clarify the positional relationship of lobular and ductal carcinoma components, their histological distributions were examined precisely in 14 combined cases. Case no. 15 was not available for post-operative examination and was, therefore, excluded. In 13 of 14 cases, the lobular and ductal carcinoma components coexisted in the same area and were contiguously distributed. A representative case of contiguous distribution is shown in Fig. 2(a,c). LCIS and DCIS coexisted and partially overlapped each other within a partially resected breast. The only separate case (Case no. 11) is shown in Fig. 2(b). In this case, ILC and DCIS were located separately in opposite sides across the nipple in a completely resected breast.

### Clonal analysis

**Figure 3:**
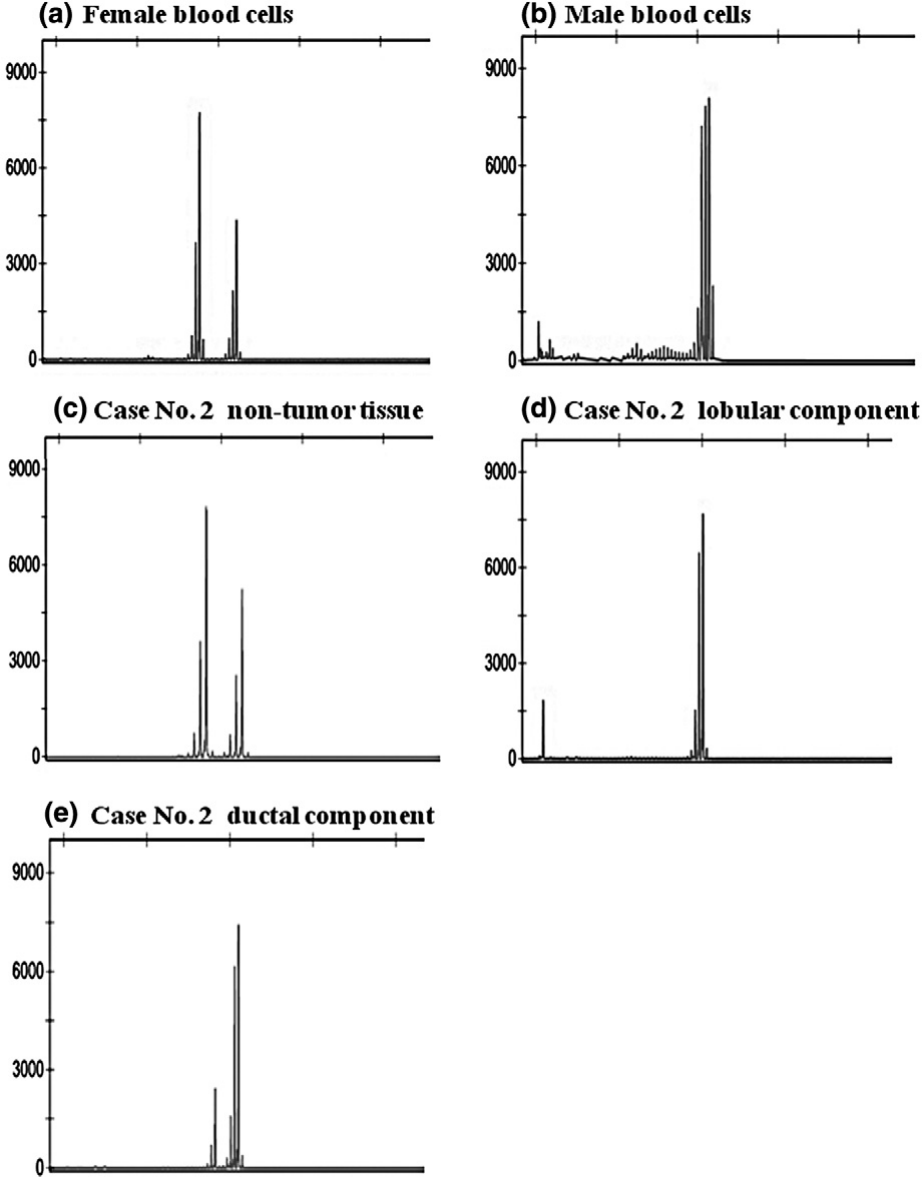
Clonal analysis with X-chromosome inactivation patterns by methylation-specific PCR on the *HUMARA* (human androgen receptor) gene. The bisulfite-treated DNA was amplified with PCR in use of methylated and unmethylated primer pairs. (a) A random X chromosome inactivation (heterozygous) pattern is shown in control female blood cells. (b) In contrast, a non-random inactivation (homozygous) pattern is shown in male blood cells. (c) The PCR products of case no. 2 are displayed as a random inactivation (heterozygous) pattern in the non-tumor tissue, and as a non-random inactivation (homozygous) pattern in both (d) the lobular carcinoma component and (e) the ductal carcinoma component. (A left-sided minor peak detected in ductal component is quite less dominant than the main peak and accessed as an artifact.)

The cellular clonal analysis was performed using a methylation-specific PCR on the *HUMARA* gene. Results are presented as a peak image in Fig. 3. The horizontal axis indicates the length of PCR products in base pairs, and the amount of fluorescence-labeled PCR products correlates to the height of the peaks. As a control, blood cells from a healthy heterozygous female were examined, and the PCR products were detected as two peaks, corresponding to the inactivated maternal or paternal X chromosome with methylated primer pairs (Fig. 3a). Activated maternal or paternal X chromosome with unmethylated primer pairs showed the same peaks. In contrast, male blood cells have only one X-chromosome, which is used as a homozygous or monoclonal control. The PCR products were detected as a non-random inactivation pattern, that is, as one peak (Fig. 3b). All 15 combined cases were examined, and the results are summarized in Table 4. The PCR products were yielded from both lobular and ductal carcinoma components in 9 out of 15 cases, and exhibited non-random X chromosome inactivation patterns, that is homozygosity suggesting monoclonality. In the other 6 cases the PCR products were unable to be determine possibly due to the DNA damage in formalin-fixed paraffin-embedded tissue or with bisulfate treatment. Representative image data of combined case no. 2 which has LCIS as a lobular component and DCIS as ductal component are shown in Fig. 3(c–e). The PCR products which were yielded from non-carcinomatous tissue were detected as two peaks, and were proved to be heterozygous (Fig. 3c). On the other hand, PCR products of both lobular and ductal components were detected as one peak (Fig. 3d,e). It would be interesting to see the clonal relationship between lobular and ductal carcinomas in case no. 11, since this was the only case that showed the separate distribution pattern as presented in Fig. 2(b). However, the PCR product was unfortunately not yielded from the lobular component.

**Table 4:** Results of methylation-specific PCR

	Lobular		Ductal		
Case	U	M	U	M	Results
1	a	n	n	n	
2	a	n	a	n	homozygote
3	a	n	a	n	homozygote
4	a	n	a	n	homozygote
5	a	n	a	n	homozygote
6	a	n	a	n	homozygote
7	n	n	a	n	
8	a	n	a	n	homozygote
9	a	n	a	n	homozygote
10	a	n	n	n	
11	n	n	a	n	
12	n	n	a	n	
13	a	n	a	n	homozygote
14	a	n	a	n	homozygote
15	a	n	n	n	

a, amplified; M, PCR used with methylated primer; n, not amplified; U, PCR used with unmethylated primer.

## Discussion

In this study, almost half (52%) of lobular carcinoma cases showed coexistence with ductal carcinoma in the ipsilateral breast, designated as combined lobular and ductal carcinoma. The rate of 52% is higher than the previous reports (5–15%).^9^–^13^ More than half (60%) of the combined cases comprised the LCIS as a lobular component. Even if LCIS were excluded in this study, because most previous reports did not comprise LCIS, the rate of combined cases will be 30%, which is still higher than the previous studies. It is highly likely that immunostaining of E-cadherin and β-catenin increased the detection rate of small lobular lesions in this study, resulting in the higher percentage of combined lobular carcinoma. Clinicopathologically, combined lobular cases were younger than pure lobular cases, and the average tumor size of lobular component of combined cases was smaller than that of pure lobular cases. In combined cases, the cytological atypia of lobular component, which comprised LCIS, was low (grade 1). These tendencies suggest that combined lobular carcinomas could be detected in the earlier stage. On the other hand, pure lobular cases were of all invasive carcinoma, although this is partly caused by the clinical difficulty of detecting LCIS without abnormal signs in the mammogram.^30^,^31^ It is noteworthy that lobular and ductal components coexisted in the neighborhood and were distributed contiguously. The immunohistochemical phenotypes of both components were accorded in most combined cases (93%). In an exceptional case (no. 3), the lobular component was ER+/PgR-/HER2-, whereas the ductal component was ER+/PgR-/HER2-. Although the expression of PgR is under control of ER gene, the expression level of PgR may not necessarily be in agreement with ER. The intrinsic subtypes of breast cancer of both ER+/PgR-/HER2- and ER+/PgR-/HER2- lead to the same luminal A type,^19^ and this immunohistochemical inconsistency does not mean genetic heterogeneity or polyclonal.

**Figure 4:**
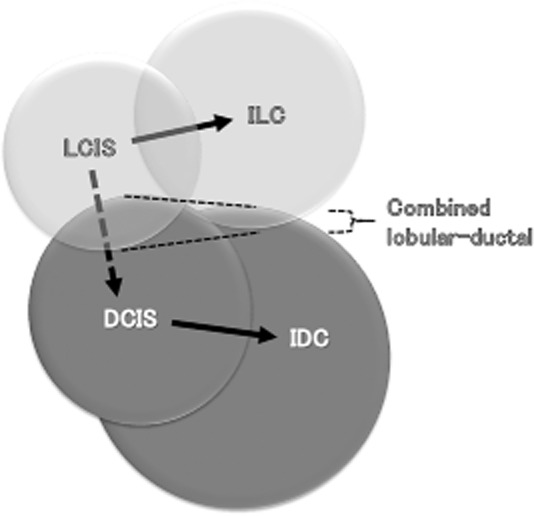
Histogenesis of combined lobular and ductal carcinoma derived from LCIS as a common precursor lesion. LCIS, lobular carcinoma in situ; DCIS, ductal carcinoma in situ; ILC, invasive lobular carcinoma; IDC: invasive ductal carcinoma.

These do not suggest that lobular and ductal components could arise independently and collide as traditionally believed. It is proposed that the lobular and ductal components could arise from a common precursor lesion, which would be LCIS, because of the predominance (Fig. 4). In order to prove this hypothesis, a clonal analysis using methylation-specific PCR on the *HUMARA* gene was undertaken. It demonstrated that the same allele was inactivated in both lobular and ductal components in all nine detectable cases. On the basis of genetic rule, inactivation of each maternal or paternal allele of individual breast cells occurs evenly in the embryogenesis prior to the carcinogenesis. Inactivation of the same allele of each lobular and ductal component of combined type could occur with a probability of 1/2, if they derived from the different cells.^32^ If it happened to be inactivated in the same allele of each components in 9 cases, the probability becomes (1/2)^9^ = 1/512. Therefore, it is reasonable to assume that both lobular and ductal components of combined cases derived from the LCIS as the common precursor lesion of TDLU.

In summary, the detailed immunohistochemical study revealed that combined lobular and ductal carcinomas were frequently observed in the early stages. The lobular and ductal components of combined carcinoma coexist in the marked proximity. Furthermore, they were proven to be statistically clonal by methylation-specific PCR analysis on the *HUMARA* gene. These indicate that LCIS could be a putative precursor for the subsequent development of invasive carcinoma of either lobular or ductal type.
